# Familial hemiplegic migraine Ca_V_2.1 channel mutation R192Q enhances ATP-gated P2X_3 _receptor activity of mouse sensory ganglion neurons mediating trigeminal pain

**DOI:** 10.1186/1744-8069-6-48

**Published:** 2010-08-24

**Authors:** Asha Nair, Manuela Simonetti, Nicol Birsa, Michel D Ferrari, Arn MJM van den Maagdenberg, Rashid Giniatullin, Andrea Nistri, Elsa Fabbretti

**Affiliations:** 1 Neurobiology Sector, International School for Advanced Studies (SISSA), Via Bonomea 265, 34136 Trieste, Italy; 2 Leiden University Medical Centre, Department of Neurology, 2300 RC Leiden, The Netherlands; 3 Leiden University Medical Centre, Department of Human Genetics, 2300 RC, Leiden, The Netherlands; 4 Department of Neurobiology, A. I. Virtanen Institute, University of Eastern Finland, 70211 Kuopio, Finland; 5 University of Nova Gorica, SI-5000, Slovenia; 6 Current Address: Institute for Molecules and Materials, University of Nijmegen, Toernooiveld 1, Nijmegen 6525 ED, The Netherlands; 7 Current Address: Pharmacology Institute, Faculty of Medicine, University of Heidelberg, Im Neuenheimer Feld 366, 69120 Heidelberg, Germany

## Abstract

**Background:**

The R192Q mutation of the CACNA1A gene, encoding for the α1 subunit of voltage-gated P/Q Ca^2+ ^channels (Ca_v_2.1), is associated with familial hemiplegic migraine-1. We investigated whether this gain-of-function mutation changed the structure and function of trigeminal neuron P2X_3 _receptors that are thought to be important contributors to migraine pain.

**Results:**

Using in vitro trigeminal sensory neurons of a mouse genetic model knockin for the CACNA1A R192Q mutation, we performed patch clamp recording and intracellular Ca^2+ ^imaging that showed how these knockin ganglion neurons generated P2X_3 _receptor-mediated responses significantly larger than wt neurons. These enhanced effects were reversed by the Ca_v_2.1 blocker ω-agatoxin. We, thus, explored intracellular signalling dependent on kinases and phosphatases to understand the molecular regulation of P2X_3 _receptors of knockin neurons. In such cells we observed strong activation of CaMKII reversed by ω-agatoxin treatment. The CaMKII inhibitor KN-93 blocked CaMKII phosphorylation and the hyperesponsive P2X_3 _phenotype. Although no significant difference in membrane expression of knockin receptors was found, serine phosphorylation of knockin P2X_3 _receptors was constitutively decreased and restored by KN-93. No change in threonine or tyrosine phosphorylation was detected. Finally, pharmacological inhibitors of the phosphatase calcineurin normalized the enhanced P2X_3 _receptor responses of knockin neurons and increased their serine phosphorylation.

**Conclusions:**

The present results suggest that the CACNA1A mutation conferred a novel molecular phenotype to P2X_3 _receptors of trigeminal ganglion neurons via CaMKII-dependent activation of calcineurin that selectively impaired the serine phosphorylation state of such receptors, thus potentiating their effects in transducing trigeminal nociception.

## Background

Migraine is a common debilitating neurovascular disorder with complex etiology that is clinically divided into two main subtypes based on the absence or presence of an aura that is characterized by transient visual, sensory and/or speech related neurological symptoms [1]. Most molecular genetic insight in the pathophysiology of migraine comes from studies of a rare monogenic subtype of migraine with aura, called Familial Hemiplegic Migraine type 1 (FHM-1) [2]. FHM-1 is due to mutations in the α1 subunit of voltage-gated Ca_V_2.1 (P/Q-type) Ca^2+ ^channels [3]. Transgenic knock-in (KI) mice carrying a FHM-1 glutamine for arginine (R192Q) mutation revealed increased glutamate release in the cortex that explains their increased susceptibility to cortical spreading depression (CSD) [4,5], the electrophysiological correlate of the human migraine aura [6]. Patch clamp analysis of transfected neurons expressing human R192Q-mutant α1 protein revealed facilitated activation of Ca_V_2.1 channels and increased Ca^2+ ^influx as the underlying molecular mechanism [7]. Whereas there is little doubt that CSD causes the aura, the mechanisms leading migraine headache are less clear. The current view is that the headache is caused by the trigeminovascular system that releases a number of "migraine mediators" activating nociceptive receptors expressed by trigeminal neurons [8]. Previous studies from our group have indicated that migraine mediators (e.g., nerve growth factor and calcitonin gene-related peptide) can persistently sensitize trigeminal sensory neurons [9-11] and that enhanced pain perception involves activation of neuronal P2X_3 _receptors by extracellular ATP [12]. The role of trigeminal sensory neuron P2X_3 _receptors as important contributors to migraine pain has recently been discussed [13-16].

The present study used R192Q KI mice to investigate peripheral pain mechanisms at the level of trigeminal sensory neurons to explore whether they show evidence of enhanced pain transduction. Since Ca_V_2.1 Ca^2+ ^channels normally are expressed by trigeminal sensory neurons and contribute by approximately 40% to voltage-gated Ca^2+ ^influx [17], we assessed whether the R192Q mutation might introduce a cascade of Ca^2+^-dependent signals controlling expression and function of the main pain receptors P2X_3 _and TRPV1 [18-21]. Although we observed unchanged activity of TRPV1 receptors in mutant mouse sensory neurons, we detected a significant increase in P2X_3 _receptor activity that was characterized by combining electrophysiological and molecular biology techniques.

## Results

### Ca_V_2.1 R192Q KI trigeminal neurons show enhanced membrane currents mediated by P2X_3 _receptors

**Figure 1:**
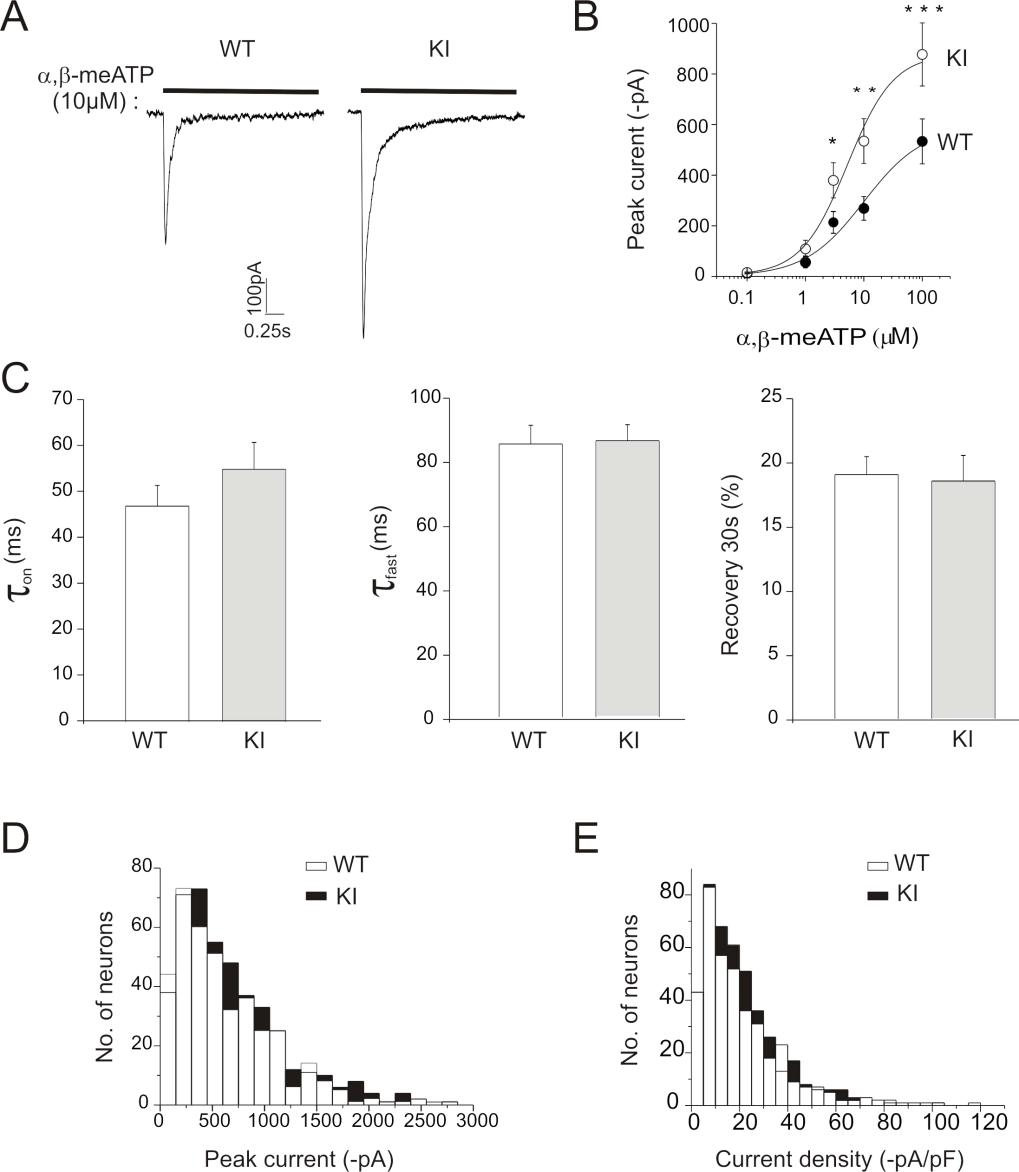
**Functional characterization and expression of P2X_3 _receptors in WT and R192Q KI trigeminal ganglion neurons**. *A*, Representative current traces induced by α,β-meATP (10 *μ*M, 2-s application) on WT and KI neurons. *B*, Dose-response curves for WT (*n *= 18, *filled circles*) and KI (*n *= 17, *open circles*) cells. * *p *= 0.04, ** *p *= 0.035, ***p = 0.03. Note larger responses of α,β-meATP-mediated currents on KI neurons with respect to WT, as the dose-effect plot is shifted upwards for KI neurons. *C*, Rise time (*left*; expressed as τ_on _calculated on the 10-90% response rise), desensitization onset (*middle*; expressed as the first time constant, τ_fast_, of current decay) and recovery from desensitization (*right*; expressed as% of control amplitude in a paired pulse agonist application) of P2X_3 _receptor currents are similar for WT and KI neurons. All responses were evoked by α,β-meATP (10 μM, 2 s). *n *= 154 for WT and *n *= 183 for KI. *D*, Histograms show peak amplitude distribution of α,β-meATP (10 *μ*M)-induced P2X_3 _currents for WT (*n *= 414, *open bars*) and KI (*n *= 454, *filled bars*). *E*, Histograms show the distribution of current density (i.e., current amplitude normalized with respect to the neuronal capacitance, pA/pF) for WT (*n *= 414, *open bars*) and KI (*n *= 454, *filled bars*) neurons, indicating significantly higher α,β-meATP-evoked KI responses over a span of 15-35 pA/pF values compared to WT.

Because the large majority of trigeminal ganglion neurons typically express P2X_3 _receptors [22] that are implicated in pain transducing mechanisms in migraine [8], we investigated whether the Ca_V_2.1 channel α1 subunit R192Q mutation affected P2X_3 _receptor function. Fig. 1A shows examples of current traces induced by a 2-s application of the selective P2X_3 _receptor agonist α,β-meATP (10 *μ*M) to WT and R192Q KI neurons. On WT neurons, the fast-developing inward current was on average -354 ± 28 pA (*n *= 154) and then fully desensitized during agonist application, a characteristic typical of currents mediated by P2X_3 _receptors [18]. On R192Q KI neurons, the peak amplitude of α,β-meATP-evoked current was, on average, -547 ± 32 pA (*n *= 183: *p *= 0.035 from WT) with subsequent full desensitization. Fig. 1B shows average concentration-response plots for WT (*n *= 18) and R192Q KI (*n *= 17) neurons tested with α,β-meATP. The R192Q KI plot revealed a significantly larger maximal response compared to WT without changing the α,β-meATP potency (EC_50 _= 6.9 ± 1.1 *μ*M for WT; 4.3 ± 0.8 *μ*M for KI) or the Hill coefficient (0.86 ± 0.34 for WT; 1.11 ± 0.34 for KI). Other parameters of P2X_3 _receptor function, such as current rise-time (τ_on_), desensitization onset (τ_fast_), and recovery from desensitization at 30 s interpulse interval, were not significantly different between genotypes (Fig. 1C). Mouse trigeminal neurons usually express a low level of heteromeric P2X_2/3 _receptors mediating a sustained inward current following the initial transient peak [22]. In the present study no significant difference in the amplitude or occurrence of heteromeric responses (measured at steady state) was observed between WT and KI neurons (-60 ± 6 pA in WT, *n *= 53; 34% incidence and -61 ± 8 pA in KI, *n *= 47; 25% incidence).

**Figure 2:**
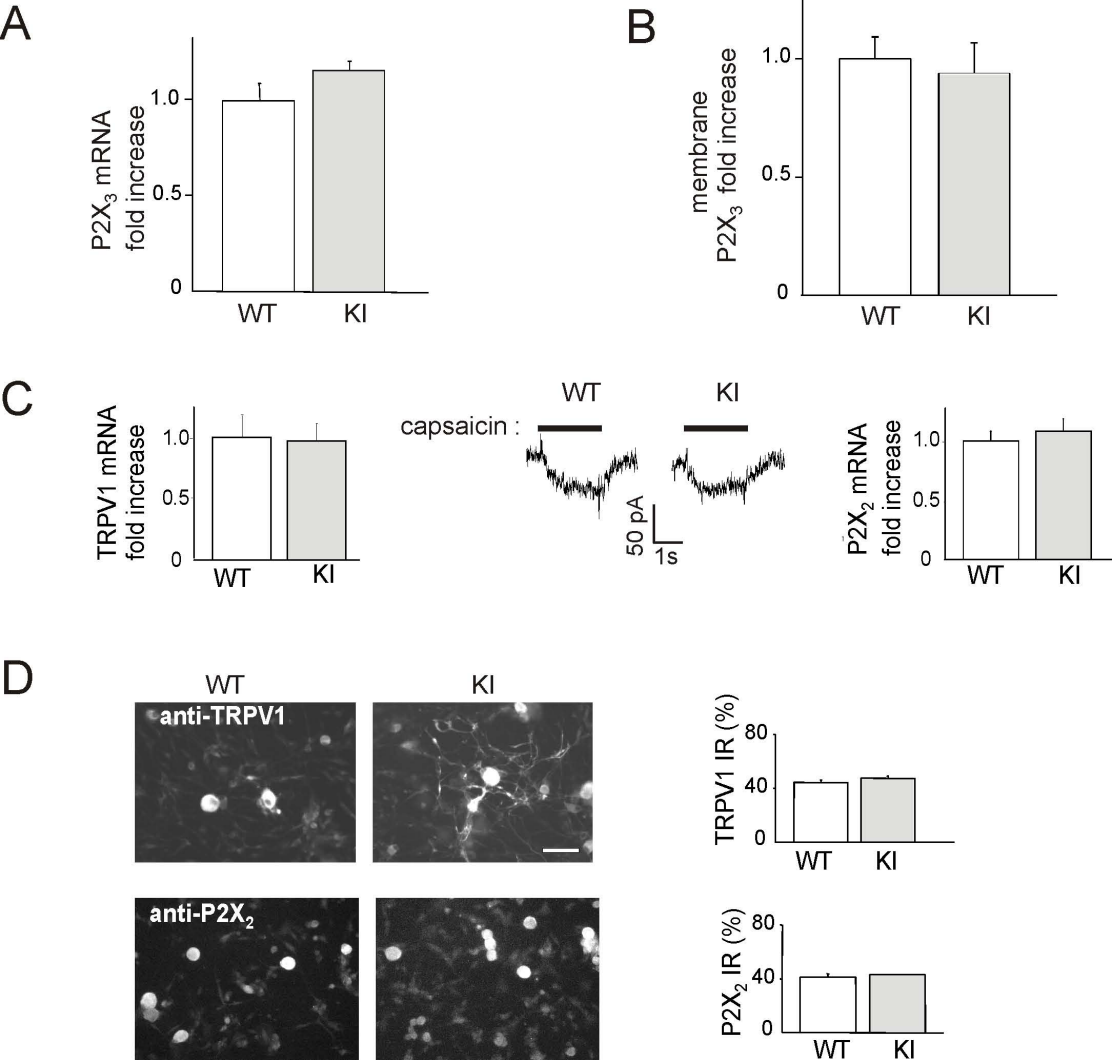
**Expression of pain receptors in WT and KI neurons**. *A*, Real-time RT-PCR experiments of mRNA extracts from trigeminal ganglia show no significant difference in P2X_3 _mRNA expression levels between WT and KI mice (*n *= 3, *p *> 0.05). Data were normalized with respect to β-tubulin III housekeeping mRNA content. *B*, Biotinylation experiments of trigeminal neurons in culture show no significant differences in P2X_3 _membrane protein expression levels between WT and KI mice (*n *= 4, *p *> 0.05). *C*, Real time RT-PCR experiments to measure mRNA expression levels of TRPV1 (left) and P2X_2 _(right) receptor subunits show no significant difference between trigeminal ganglia from WT and KI mice. *n *= 3 *p *> 0.05. Middle panel shows examples of TRPV1 receptor mediated responses evoked by capsaicin (1 μM) from WT or KI neuron, indicating similar amplitude and duration. *D*, Microphotographs show immunoreactivity of TRPV1 and P2X_2 _proteins in cultured trigeminal neurons from WT or KI mice. Histograms (*right*) represent% of immunoreactive neurons for TRPV1 (*top*) and P2X_2 _(*bottom*) over β-tubulin III immunoreactive neurons. *n *= 5, *p *> 0.05.

Fig. 1D,E shows the amplitude distribution of current responses evoked by α,β-meATP (10 *μ*M) in WT and R192Q KI neurons (*n *= 454 and *n *= 414, respectively). Because of the heterogeneity of trigeminal sensory neurons in culture, responses were further analyzed in terms of cell capacitance, which reflects the cell size. In view of the distribution of P2X_3 _receptor expression [22], we restricted our sampling to neurons that are larger than 15 *μ*m in diameter. Fig. 1E shows that enhanced responses to α,β-meATP were observed in a population of neurons with 15-35 pA/pF current density value that matched the somatic size profile shown in Fig. 1D. This upregulation of P2X_3 _receptor function was not due to increased receptor synthesis and/or expression at membrane level as indicated by real time RT-PCR data (Fig. 2A) and membrane biotinylation results (Fig. 2B; *p *> 0.05) performed in accordance with our previous studies [9,10]. Current responses induced by 1 *μ*M capsaicin, a selective agonist of TRPV1 channels, were not significantly different between WT (-38 ± 3 pA; *n *= 64) and KI (-40 ± 4 pA; *n *= 80) neurons as exemplified by the middle panel of Fig. 2C. mRNA synthesis and immunohistochemical expression of TRPV1 and P2X_2 _receptors was similar between WT and KI (Fig. 2C,D).

The enhanced responses of P2X_3 _receptors on KI neurons were probably related to the Ca_V_2.1 R192Q channel activity because pre-treatment of KI cultures with the selective inhibitor ω-agatoxin (200 nM; 30 min) produced KI neuronal responses to α,β-meATP that were 52 ± 4.5% (*n *= 17) of untreated KI (*n *= 19; *p *= 0.001). Pretreatment of WT neurons with ω-agatoxin induced only a relatively minor decrease in amplitude of P2X_3 _currents (71 ± 3.5%; *n *= 10 vs. untreated WT cells taken as 100%; *n *= 14).

### Intracellular Ca^2+ ^transients of Ca_V_2.1 R192Q neurons

**Figure 3:**
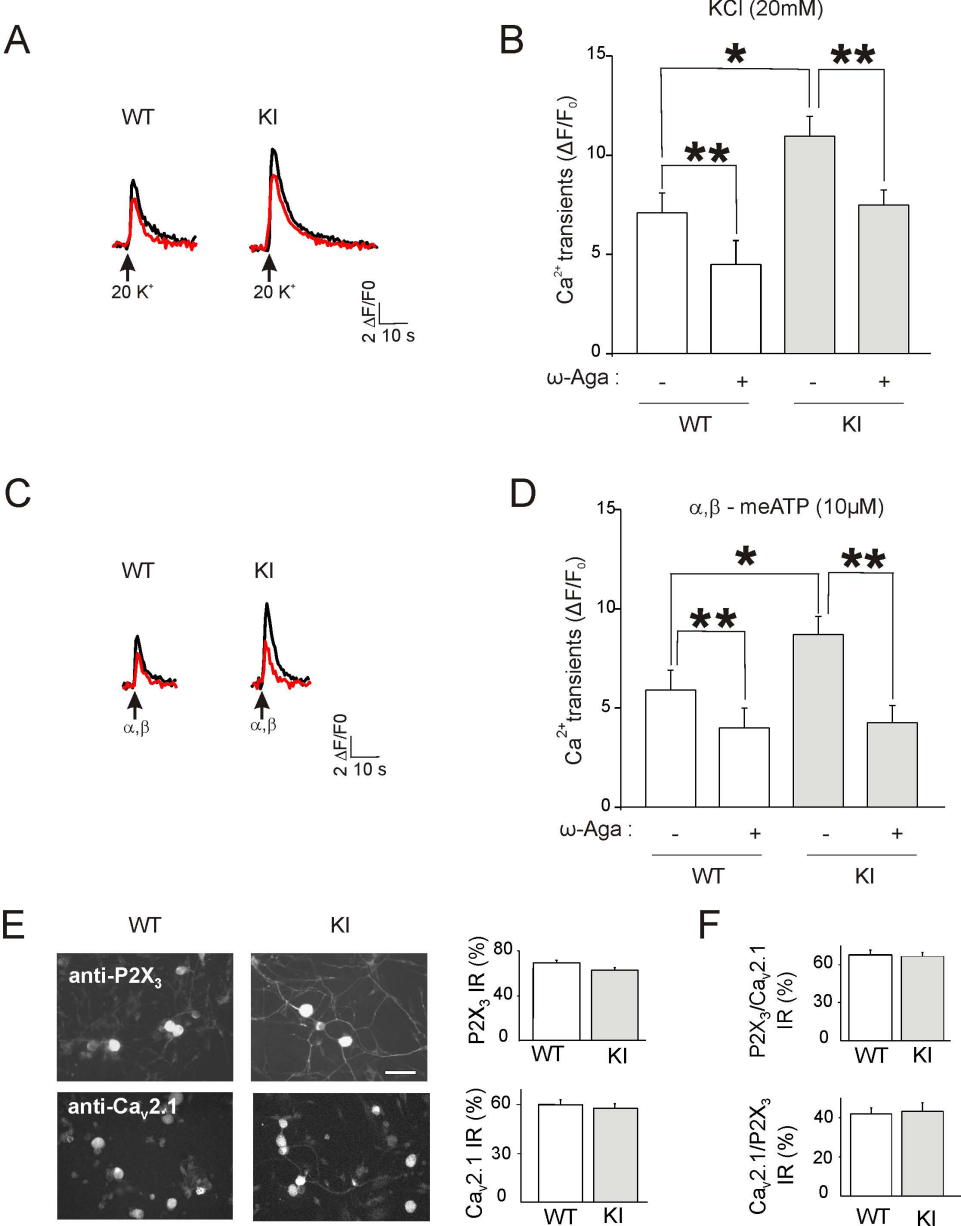
**Ca^2+ ^transients evoked by K^+ ^depolarization or P2X_3 _receptors in WT and R192Q KI neurons**. *A*, Examples of Ca^2+ ^transients of trigeminal neurons evoked by KCl (20 mM, 2-s application) before (*black trace*) and after (*red trace*) application of ω-agatoxin (200 nM, 30 min). *B*, KI neurons show significant increase in KCl (20 mM, 2-s application) mediated Ca^2+ ^transients compared to WT (*p = 0.005, *n *= 28 and *n *= 45, in WT and KI, respectively). Histograms also represent inhibition by ω-agatoxin of Ca^2+ ^transients for WT (*n *= 14) and KI (*n *= 35) neurons. After ω-agatoxin responses of WT and KI neurons differ from their own controls (**p ≤ 0.001). *C*, Representative traces of α,β-meATP (10 *μ*M, 2-s application)-evoked Ca^2+ ^transients before (*black trace*) and after (*red trace*) application of ω-agatoxin (200 nM, 30 min). *D*, Histograms show larger Ca^2+ ^transients evoked by α,β-meATP (10 *μ*M, 2-s application) from KI (*n *= 26) than WT (*n *= 16) and neurons (*p = 0.04). Histograms also show that ω-agatoxin reduced Ca^2+ ^transients of KI (*n *= 22) and WT (*n *= 9) neurons. **p ≤ 0.001 for each case. *E*, Microphotographs of immunofluorescence experiments depicting WT and KI trigeminal neurons in culture expressing P2X_3 _receptors or Ca_V_2.1 channels. *Bar *= 50 *μ*m. Histograms (*right*) show% of P2X_3_- (top) or Ca_V_2.1- (bottom) immunoreactive neurons (taking as 100% the β-tubulin III immunoreactive) (*n *= 5, *p *> 0.05 for P2X_3 _receptors; *n *= 3, *p *> 0.05 for Ca_V_2.1-expressing neurons). *F*, Histograms show% of Ca_V_2.1-immunoreactive neurons (*top*; taken as 100%) which are immunopositive for P2X_3 _(*n *= 7, *p *> 0.05) or% of P2X_3_-immunoreactive neurons (*bottom*) which are immunopositive for Ca_V_2.1 *(n *= 4, *p *> 0.05).

Without perturbing intracellular Ca^2+ ^homeostasis with the whole-cell patch clamp method, we tested the contribution of P/Q-type channels to intracellular Ca^2+ ^levels following depolarizing pulses of K^+ ^or α,β-meATP. This approach enabled us to test the excitability (in terms of intracellular Ca^2+ ^transients) of trigeminal sensory neurons [10]. To this end, we compared Ca^2+ ^transients evoked by these stimuli before and after 30 min application of ω-agatoxin (200 nM) to WT or KI neurons. It is noteworthy that the size of the intracellular Ca^2+ ^rise induced by α,β-meATP was significantly smaller than the one elicited by K^+ ^presumably because the amplitude of neuronal membrane depolarization (and its duration) evoked by K^+ ^was much larger and, thus activated a wider population of Ca^2+ ^channels [10,22]. This finding is corroborated by the data shown in Fig. 3A.

Despite comparable levels of dye loading and similar responses to rather high (50 mM) pulse application of K^+ ^(not shown), we consistently found that KI neurons produced significantly (*p = *0.005) larger Ca^2+ ^transients (Fig. 3B) when stimulated with a lower concentration (20 mM) of K^+^. We also observed that 78% of KI neurons (*n *= 35/45) were sensitive to ω-agatoxin inhibition (i.e., showed a response decrease of >10%), whereas in the case of WT neurons, only 50% (*n *= 14/28) were sensitive to this toxin. Fig. 3B indicates that, following application of ω-agatoxin, the depression of Ca^2+ ^transients induced K^+ ^was proportionally similar in WT and KI neurons (27 ± 4%, *n *= 14; and 30 ± 3%, *n *= 35, respectively), indicating that the toxin-sensitive contribution to the global Ca^2+ ^transient was not affected by the Ca_V_2.1 mutation that is reported to affect only the activation threshold of P/Q-type channels [4,5].

In line with these results, we also observed that Ca^2+ ^transients induced by α,β-meATP (10 *μ*M) on KI neurons were larger (*p *= 0.04) than on WT neurons (Fig. 3C,D), although a similar percentage of WT (47%) and KI (46%) neurons were sensitive to α,β-meATP. On the same neurons tested before and after 30 min application of ω-agatoxin (200 nM), α,β-meATP-evoked Ca^2+ ^responses were inhibited in 56% WT and 85% KI neurons. Fig. 3D demonstrates that, on average, ω-agatoxin significantly (*p *< 0.001) depressed such Ca^2+ ^transients of KI or WT neurons with respect to untreated cells. Furthermore, the decrease in Ca^2+ ^transient amplitude by ω-agatoxin was significantly (*p *= 0.04) larger (43 ± 2%, *n *= 22) for KI neurons than for WT neurons (30 ± 2%, *n *= 9). Nevertheless, after ω-agatoxin, the residual Ca^2+ ^response to α,β-meATP was similar for WT and KI neurons (see Fig. 3D) probably because the size of the depolarization elicited by α,β-meATP was not large enough to activate a major fraction of the high threshold Ca^2+ ^channels [22]. Thus, this residual response was likely made up by the analogous Ca^2+ ^permeability through P2X_3 _receptor channels of WT and KI neurons.

Immunohistochemical analysis of trigeminal ganglion cultures showed that the number of immunopositive neurons for Ca_V_2.1 channels and P2X_3 _receptors was similar between genotypes (Fig. 3E). We also investigated (by double immunofluorescence with anti-P2X_3 _and anti-Ca_V_2.1 antibodies) the fraction of Ca_V_2.1 immunoreactive neurons expressing P2X_3 _receptors as well: this number amounted to 68 ± 4% for WT and 66 ± 3% for KI (*n *= 7, *p *> 0.05) for small-medium neurons (<20 μm), suggesting a large, though not complete, occurrence of protein co-expression. In fact, when we examined the number of P2X_3 _immunoreactive neurons that were positive for Ca_V_2.1, we found 42 ± 3% and 43 ± 4% for WT and KI, respectively (*n *= 4). These observations were consistent with a functional interaction between these proteins in a subpopulation of sensory neurons.

### High CaMKII activity confers enhanced P2X_3 _receptor phenotype to KI trigeminal neurons

**Figure 4:**
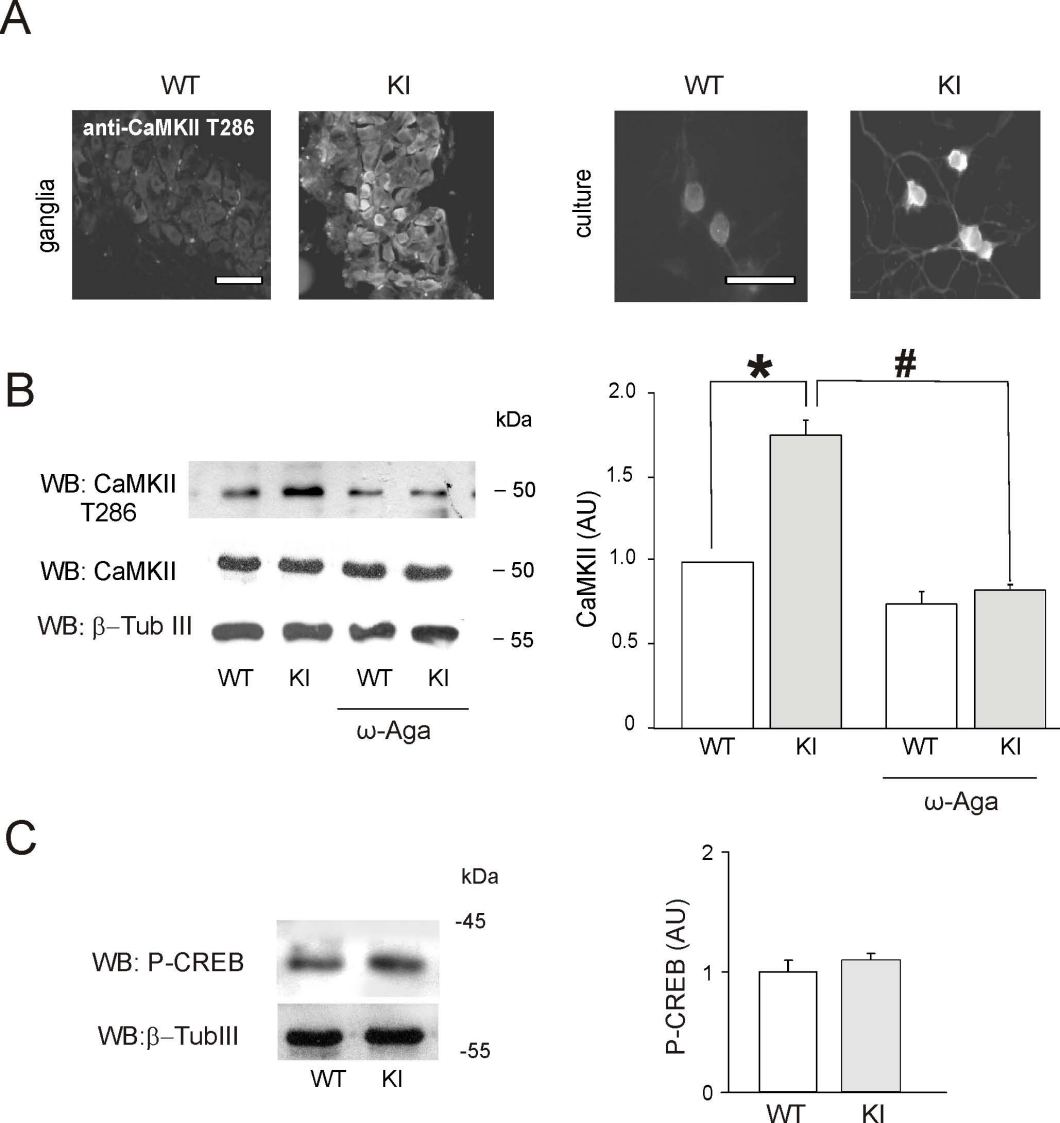
**CaMKII activation of R192Q KI trigeminal neurons is reversed by ω-agatoxin**. *A*, Microphotographs of immunofluorescence experiments with anti-phospho Thr286 CaMKII antibody (recognizing the active form of CaMKII) of WT and KI trigeminal ganglia (*left panel*) or trigeminal neurons in culture (*right panel*). *Bar *= 50 *μ*m. *B*, Example of western immunoblots of total protein lysate from WT and KI trigeminal neurons in culture using antibodies recognizing the active form of CaMKII (phosphorylated Thr286, *upper lanes*) or anti-total CaMKII antibody (*middle lanes*). Equal loading was tested with β-tubulinIII antibodies (*bottom lanes*). *p *= 0.03, (*n *= 4). CaMKII activation is prevented by treatment with ω-agatoxin (200 nM, 24 h). Histograms (*right*) demonstrate significant increase in active (threonine-phosphorylated) CaMKII in KI neurons, an effect prevented by ω-agatoxin (*n *= 4, #*p *= 0.027). *C*, Western immunoblot experiments of trigeminal ganglia from WT and KI mice, using the same anti-phospho-CREB antibody (43 kDa). Gel loading quantification is obtained using anti-β-tubulinIII antibody. Histograms (*right*) show no significant difference in CREB phosphorylation between the two conditions. *n *= 3, *p *> 0.05.

**Figure 5:**
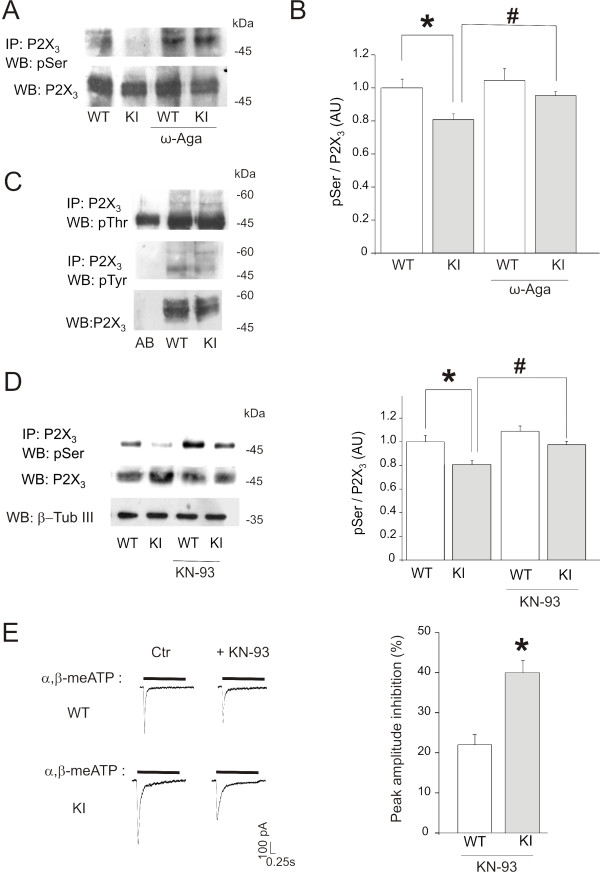
**Phosphorylation state of P2X_3 _receptors in KI neurons**. *A*, Example of western blots of P2X_3 _receptors immunopurified from WT and KI neurons and probed with anti-phosphorylated serine antibodies. Note decreased serine phosphorylation of KI samples, a phenomenon prevented by pre-treatment with ω-agatoxin (200 nM, 24 h). *B*, Histograms quantify serine phosphorylation state of P2X_3 _receptors (*p = 0.02 vs. WT; *n *= 5) that is enhanced for KI receptors and prevented by ω-agatoxin (*n *= 3, #*p *= 0.027). *C*, Example of western blots of immunopurified P2X_3 _receptors from WT and KI neurons probed with anti-phosphorylated threonine (top row) or tyrosine (middle row) antibodies. Total P2X_3 _receptor levels are shown in bottom row. Control antibody lane (Ab) is also shown. *n *= 8 for threonine and *n *= 3 for tyrosine. *D*, Pretreatment of neurons with the CaMKII inhibitor KN-93 (5 *μ*M, 90 min) restores the P2X_3 _serine phosphorylation state without changing total P2X_3 _receptor expression. Histograms (*right*) quantify serine phosphorylation state of P2X_3 _receptors, an effect blocked by KN-93 (*n *= 3; *p = 0.02; #*p *= 0.017) in KI neurons. *E*, Representative examples of WT and KI current traces evoked by α,β-meATP (10 *μ*M, 2-s application) in control (Ctr) and after pre-treatment with KN-93 (5 *μ*M, 90 min). Histograms (*right*) show the percent inhibition by KN-93 of peak amplitude of α,β-meATP-mediated P2X_3 _receptor currents in KI neurons (*n *= 22), that is significantly larger in KI cells (*n *= 17, *p = 0.039).

To discover the mechanisms responsible for the enhanced activity of trigeminal P2X_3 _receptors despite no change in their expression levels, we studied whether the molecular regulation of the receptors was changed by the R192Q gain-of-function mutation of Ca_V_2.1 channels. Our previous studies have demonstrated that changes in P2X_3 _receptor activity could be brought about by pharmacological manipulations affecting the phosphorylation state of the receptor, rather than its expression level [9,23,12]. Thus, it seemed likely that the origin of the augmented P2X_3 _receptor activity of KI neurons might reside in a changed phosphorylation state of the intracellular protein domains. It is likely that increased Ca^2+ ^influx via mutated Ca_V_2.1 channels may regulate multiple intracellular Ca^2+^-dependent signalling mechanisms via altering the physiological function of many Ca^2+ ^binding proteins, amongst which CaMKII is a likely contributor [24,25]. For these reasons, immunofluorescence experiments were performed with antibodies selective for active CaMKII phosphorylated at Thr286 [11] that showed a larger number of immunopositive trigeminal neurons in culture (45 ± 5%), and intact ganglia (36 ± 6%) from KI mice (Fig. 4A). By comparison, CaMKII immunoreactivity was detected only in 15 ± 4 and 9 ± 3% of neurons in cultures or intact ganglia from WT mice (*n *= 3-5; *p *= 0.03; see example in Fig. 5A). Previous experiments have confirmed the antibody selectivity by showing that its signal was blocked by the CaMK inhibitor KN-93 in immunofluorence and western blotting [11].

Immunoblotting performed on protein extracts from trigeminal ganglia and cultures further demonstrated that, despite the lack of change in total CaMKII level between WT and KI (*n *= 4, *p *> 0.05; Fig. 4B), the active fraction of CaMKII (T286-phosphorylated) was significantly larger in KI than WT (Fig. 4B; *n *= 4, *p *= 0.03). We also observed that the larger activation of CaMKII in KI samples was due to the presence of mutated Ca_V_2.1 channel activity, because pretreatment with ω-agatoxin (200 nM, 24 h) significantly (*p *= 0.027; *n *= 4) reduced the CaMKII signal (Fig. 4B). Increased CaMKII activity in KI neurons was not apparently accompanied by an overactivation of the CREB transcription factor compared with that in WT neurons (Fig. 4C, see [11]).

### R192Q KI neurons lack constitutive serine phosphorylation of P2X_3 _receptors

The observation of strong CaMKII activation in KI neurons led us to examine the phosphorylation state of their P2X_3 _receptors. Hence, we screened immunoprecipitated P2X_3 _receptors from WT and KI trigeminal neurons with antibodies against phosphorylated residues. Fig. 5A,B shows that P2X_3 _receptor serine-phosphorylation was significantly reduced in extracts of trigeminal cultures (*n *= 5, *p *= 0.02) from KI compared with WT. Although significant, this difference was not very large probably because the number of P2X_3 _receptor expressing neurons that also expressed Ca_V_2.1 channels was less than 50% of the total population whose contribution diluted the overall change (see Fig. 3F). Notwithstanding this issue, the dissimilar level of P2X_3 _receptor serine-phosphorylation was also found between KI and WT ganglia (*n *= 3, *p *= 0.04; data not shown). It is noteworthy that the antibodies we used for studying the phosphorylated aminoacid residues were not sequence-specific and, therefore, did not allow us to identify the consensus sequence of the receptor domain responsible for conferring the upregulated phenotype of KI neurons.

When cultures were preincubated with ω-agatoxin (200 nM, 24 h), P2X_3 _serine phosphorylation was significantly (*p *= 0.027) increased in KI neurons (*n *= 3; Fig. 5A). We observed no significant change in threonine (*n *= 5) or tyrosine phosphorylation level (*n *= 5, *p *> 0.05; Fig 5C). These results suggest that R192Q mutation in Ca_V_2.1 channels conferred to KI trigeminal neurons a molecular phenotype with constitutively depressed serine phosphorylation of P2X_3 _receptors, together with upregulation of P2X_3 _receptor-mediated currents.

The change in P2X_3 _receptor serine phosphorylation of KI neurons was linked to high CaMKII activity because pre-application of the selective CaMKII inhibitor KN-93 (5 *μ*M; 90 min; Fig. 5D) to KI cultures reversed this phenotype and restored a level of serine phosphorylation analogous to the WT one. Conversely, KN-93 had no effect on WT serine P2X_3 _phosphorylation probably because of the constitutively low level of CaMKII activity in these cells (see Fig. 4A,B). Patch-clamp data validated our findings because, whereas KN-93 did not significantly change membrane currents evoked by α,β-meATP (10 *μ*M) from WT neurons (*n *= 17), it significantly (*p *= 0.039) attenuated currents recorded from KI neurons (*n *= 22; Fig. 5E).

These data suggest that Ca_V_2.1 channel-mediated activation of CaMKII exerted a positive effect on P2X_3 _receptor current of KI neurons by inhibiting serine phosphorylation.

### Serine dephosphorylated P2X_3 _receptors of KI neurons show increased function

**Figure 6:**
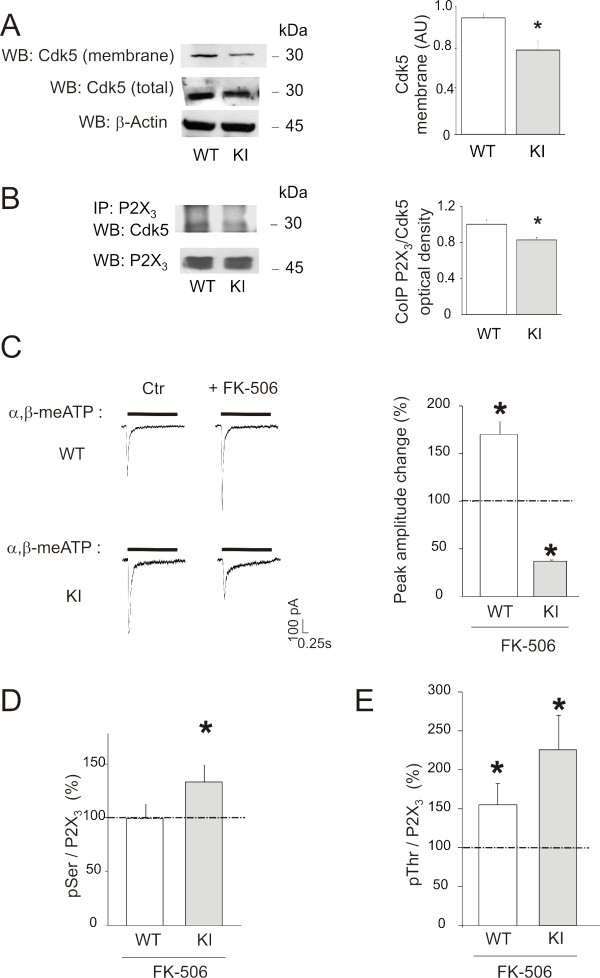
**Cdk5 and calcineurin modulate P2X_3 _receptors of trigeminal neurons**. *A*, Example of western immunoblot using anti-Cdk5 antibodies on membrane extracts from WT and KI neurons. Total Cdk5 in the soluble fraction is also shown. Actin immunoblotting ensures equal loading between WT and KI fractions (*bottom row)*. Histograms (*right*) quantify average data (*n *= 4, *p *= 0.04) of membrane Cdk5. *B*, Example of co-immunoprecipitation experiments between P2X_3 _receptors and Cdk5 in WT and KI neurons showing decreased detection of Cdk5 in P2X_3 _immunopurified samples from KI neurons. Histograms (*right*) quantify lower Cdk5 signal from KI neurons (*n *= 3, *p *= 0.03). *C*, Example of current traces recorded from WT and KI trigeminal neurons evoked by α,β-meATP (10 *μ*M, 2-s application) in control and after incubation with FK-506 (5 *μ*M, 30 min). Histograms (*right*) quantify the effect of FK-506 on the P2X_3 _receptor-mediated currents (*n *= 18, *p = 0.035 in WT neurons and *n *= 20, *p = 0.027 in KI neurons). Data are normalized to the signals from WT neurons in control conditions. Note that FK-506 inhibits P2X_3 _current responses in KI neurons. *D*, Histograms of western blot signals obtained with anti-phosphorylated serine antibody applied to immunopurified P2X_3 _receptors from WT and KI neurons in control condition and after incubation with FK-506 (5 *μ*M, 30 min). Note increase in serine phosphorylation after FK-506 treatment in KI neurons (*n *= 5, *p = 0.02). Data are normalized to the signals from WT neurons in control conditions. *E*, Histograms of western blot signals obtained with anti-phosphorylated threonine antibody show enhancement of threonine phosphorylation in WT (*n *= 5, *p = 0.046) as well as KI (*n *= 3, *p = 0.04) in neurons after treatment with FK-506. Data are normalized to the signals from WT neurons in control conditions.

We have recently observed P2X_3 _receptor serine phosphorylation by Cdk5 to be a powerful negative regulator of receptor function [26]. In keeping with the enhanced functional responses of KI neurons, we compared the expression of Cdk5 in WT and KI neurons. The percent of trigeminal neurons expressing Cdk5 was similar in WT and KI (61 ± 2%) cultured neurons (*n *= 3, *p *> 0.05). Western blotting of membrane fractions indicated a significant overall decrease of Cdk5 expression in lysates from KI ganglia (0.7 ± 0.025 fold change versus WT; *n *= 4, *p *= 0.04; Fig. 6A) together with lower P2X_3_/Cdk5 co-immunoprecipitation (0.8 ± 0.02 fold change in KI versus WT; *n *= 3, *p *= 0.03; Fig. 6B) despite analogous levels of total Cdk5 expression (see Fig. 6A), suggesting a different compartmentalisation of this molecules in KI neurons.

The observation of decreased P2X_3 _receptor serine phosphorylation in KI neurons (see Fig. 5A) prompted us to consider the potential role of phosphatases contributing to this effect. The balance between phosphorylation and dephosphorylation is a primary means for rapid regulation of a variety of neuronal functions, such as membrane excitability, neurotransmitter release, and receptor function [27]. Multiple families of serine/threonine phosphatases have been identified in the brain, including PP1, PP2A, PP2B, and PP2C families [28-30], that are closely linked to intracellular Ca^2+^. Calcineurin (PP2B) is one major Ca^2+^-dependent phosphatase that controls the activity of membrane channels [31]. Although cell-free assays show that CaMKII can inhibit calcineurin [32], in neuronal cell lines both CaMKII and calcineurin are activated by Ca^2+ ^influx and are necessary for Ca^2+^-dependent signalling pathways [33]. Hence, we tested calcineurin involvement in P2X_3 _receptor phosphorylation state and function by incubating trigeminal neurons with the calcineurin inhibitor FK-506 (5 *μ*M, 30 min). This treatment reversed the KI phenotype by decreasing the P2X_3 _receptor current (44 ± 5%, *n *= 20, *p *= 0.03; Fig. 6C) and in KI increased P2X_3 _receptor serine phosphorylation (134 ± 16%, *n *= 4, *p *= 0.03; Fig. 6D). To confirm the origin of this effect, we applied the calcineurin inhibitor peptide (100 μM) via the patch pipette during recording from WT or KI neurons. In WT neurons recorded with the peptide inhibitor filled pipettes there was a delayed (6-10 min) rise in α,β-meATP peak currents (on average -427 ± 89 pA; *n *= 8 vs - 269 ± 35; *n *= 20; *p *< 0.05), whereas for KI neurons recorded with the same protocol there was late decrease (-231 ± 28; *n *= 11 vs -477 ± 97, *n *= 8; *p *< 0.01). These experiments suggest that calcineurin activity plays an important role in regulating P2X_3 _receptor currents. Although calcineurin has been shown to facilitate desensitisation of P2X_3 _receptors expressed by oocytes [34], on native P2X_3 _receptors of mouse ganglion neurons, application of the calcineurin inhibitor FK-506 did not change the time constant of fast current decay (τ_fast_) in either WT or KI neurons (Table 1).

**Table 1:** Lack of change in desensitization onset of P2X_3 _receptors by the calcineurin inhibitor FK-506

Phenotype	**Current τ**_**fast**_	*n*
WT	81 ± 6 ms	14
WT + FK-506 (5 μM)	71 ± 7 ms	17
KI	91 ± 8 ms	18
KI + FK-506 (5 μM)	100 ± 9 ms	16

Data refers to the first time constant of current decay evoked by α,β-meATP (10 μM) taken as index of desensitization.

Application of FK-506 (5 μM, 30 min) to WT neurons did not change constitutive P2X_3 _receptor serine phosphorylation levels (*n *= 4, *p *= 0.9; Fig. 6D), significantly increased WT receptor current amplitude (170 ± 13%, *n *= 18, *p *= 0.035; Fig. 6C) and enhanced P2X_3 _threonine phosphorylation (Fig. 6E). The increase in WT receptor current amplitude in the presence of the calcineurin blocker was a phenomenon probably disjointed from serine phosphorylation state and could perhaps be attributable to increased P2X_3 _threonine phosphorylation (146 ± 22%, *n *= 5, *p *= 0.046) as previously reported for WT neurons [9].

## Discussion

The main finding of the present study is the novel report of the phenotype of trigeminal ganglion neurons from KI mice expressing a Ca_V_2.1 α1 channel subunit mutation that was previously found in FHM-1 patients [3]. KI neurons showed enhanced P2X_3 _receptor activity with decreased serine phosphorylation. This finding was associated with an increased activation of CaMKII and stronger a Ca_V_2.1 (P/Q-type) mediated rise in intracellular Ca^2+^. We envisage that such a phenotype will facilitate trigeminal pain. To the best of our knowledge, our results provide the first demonstration of P2X_3 _receptor contribution to an animal model of a neurological disease.

### Functional characteristics of the R192Q KI mouse model

ATP-gated P2X_3 _and capsaicin-sensitive TRPV1 receptors are representative of the two main classes of pain transducers in trigeminal neurons [22]. We observed potentiation of P2X_3 _receptor activity in trigeminal neurons from KI mice without changes in potency or desensitization of these receptors, and with no altered occurrence (or size) of heteromeric P2X_2/3 _responses. Likewise, no change in P2X_2 _subunit expression was detected. Responses to the TRPV1 receptor agonist capsaicin were also unaffected. Thus, our data indicate a major gain of function of P2X_3 _receptors without their concomitant overexpression at the plasma membrane or a larger number of P2X_3 _receptor expressing neurons. We set out to study whether such changes in P2X_3 _receptor function were indeed attributable to the gain of function of R192Q-mutated Ca_V_2.1 Ca^2+ ^channels in trigeminal neurons.

We showed that a subpopulation of P2X_3 _expressing neurons also expressed Ca_V_2.1 channels, thus providing ample opportunity for their crosstalk. Indeed, intracellular Ca^2+ ^imaging showed that KI neurons had stronger Ca^2+ ^transients evoked by α,β-meATP with a larger component blocked by the Ca_V_2.1 channel antagonist ω-agatoxin, indicating that this effect was probably attributable to overactivity of such mutated channels. Furthermore, ω-agatoxin reversed the mutated phenotype of P2X_3 _receptors to WT, providing a functional link between overactive Ca_V_2.1 and P2X_3 _channels. It is, however, noteworthy that in ganglia *in situ *or in culture conditions, we observed no evidence for a deleterious action by these enhanced channel activities as the number of neurons as well as the size distribution remained similar to that in WT neurons.

Hence, the present data demonstrated a novel gain-of-function produced by the R192Q mutation on P2X_3 _receptors of trigeminal ganglion neurons, that complements the synaptic upregulation reported for brain neurons [4,5].

### Molecular mechanisms involved in enhanced P2X_3 _sensitivity of R192Q KI neurons

Interestingly, KI neurons from intact trigeminal ganglia and in culture showed elevated basal activation of CaMKII (phosphorylation of its 286 threonine residue [28,35]). CaMKII is a multifunctional kinase involved in the regulation of many cell processes and its action is dependent on intracellular Ca^2+ ^changes (for review see [25]). Although we did not systematically observe spontaneous Ca^2+ ^transients in cultured trigeminal neurons, our data suggest that in KI neurons ongoing Ca^2+ ^signalling was facilitated because ω-agatoxin blocked the enhanced CaMKII activity.

We examined several intracellular targets which could be modulated by the increased CaMKII activity. Regulation of protein phosphorylation state is a common way to modify the function of pain receptors [36]. We observed that, under unchallenged conditions, the basal phosphorylation state of threonine and tyrosine in P2X_3 _receptors of KI neurons was unchanged compared with WT neurons. Likewise, there was no change in basal CREB phosphorylation. However, we did observe decreased serine phosphorylation of P2X_3 _receptors as the molecular phenotype of Ca_V_2.1-immunoreactive neurons in KI ganglia and in culture. This phenotype was strongly dependent on Ca_V_2.1 channel activity and CaMKII function, because in KI neurons, both ω-agatoxin and CaMKII inhibitor KN-93 restored the level of serine phosphorylation to that of WT, and returned P2X_3 _receptor responses to basal levels. Future experiments are necessary to identify the mechanisms regulating the basal serine phosphorylation of P2X_3 _receptors of WT cells.

### Molecular mechanisms controlling P2X_3 _receptor potentiation of KI neurons

The inverse correlation between P2X_3 _serine phosphorylation and P2X_3 _activity (i.e., reduced serine phosphorylation associated with enhanced P2X_3 _receptor function) suggests that P2X_3 _serine dephosphorylation could be an important molecular contributor to receptor operation, particularly in view of the fact that Cdk5 (a kinase associated with negative P2X_3 _receptor regulating function [26]) was less associated with P2X_3 _receptors in neuronal membranes of KI neurons.

We posited that, in WT neurons (either *in situ *or in culture), serine phosphorylation likely depended on the balance between kinases and phosphatases, in which kinases were perhaps predominant and exerting a tight regulatory role because of the consistently high basal level of serine phosphorylation we observed. On the contrary, in KI neurons such a regulation was impaired (as shown by decreased serine phosphorylation) and could be restored after treatment with calcineurin inhibitors like FK-506 [37-39] or the calcineurin peptide autoinhibitor [40]. This observation indicated that the molecular phenotype likely involved phosphatase overactivity because, when the latter was pharmacologically blocked, endogenous kinase activity readily restored serine phosphorylation. Since FK-506 is also known to inhibit ryanodine receptors to favour intracellular Ca^2+ ^leakage [39], it was useful to confirm similar observations with the calcineurin peptide autoinhibitor.

The precise molecular link between Cdk5 and calcineurin activity in terms of P2X_3 _receptor function of trigeminal neurons remains to be clarified. Upstream of this interaction, it is, however, interesting that Cdk5 is reported to downregulate P/Q-type channels [41] since in our KI model we found evidence for gain of function of such channels together with low Cdk5 membrane expression. Furthermore, Cdk5 activity is also negatively associated with activation of neuronal CaMKII [42], in analogy with the data reported in the present study. As far as the relationship between Cdk5 and calcineurin is concerned, these proteins appear to possess opposite actions on a number of intracellular neuronal proteins involved in neurotransmitter release [43,44]. Their contrasting actions are in keeping with the effects observed by us and outline even a potential process to control the release of excitatory neurotransmitter by sensory neurons, a phenomenon which might enhance trigeminal pain sensitization.

### The phosphorylation state of P2X_3 _receptors is important to control ATP-mediated responses of nociceptive neurons

Potentiation of P2X_3 _receptor responses of WT trigeminal neurons observed after application of calcineurin blockers was not accompanied by a change in receptor serine phosphorylation, that remained at its constitutive level. However, calcineurin inhibition enhanced P2X_3 _receptor threonine phosphorylation, a process that was previously associated with PKC-dependent potentiation of P2X_3 _receptor function especially after stimulation with NGF [9].

Conversely, for KI neurons, calcineurin inhibition was sufficient to reverse the depression of serine phosphorylation to a level associated with impaired receptor function despite enhanced threonine phosphorylation. Thus, our data are consistent with the notion that the phosphorylation state of serine residues is dominant to regulate P2X_3 _receptor activity.

It is well established that increased concentrations of intracellular Ca^2+ ^activate the protein phosphatase calcineurin [45,46] that is enriched in neuronal cells. We posit that the gain-of-function of mutated Ca_V_2.1 channels in Ca_V_2.1-immunoreactive neurons was accompanied by significant fluctuations in intracellular Ca^2+ ^sufficient to trigger a multisystem signaling mechanism that probably involved CaMKII and calcineurin. In addition, enhanced influx of Ca^2+ ^via mutated Ca_V_2.1 channels might modulate the activity of various transcription factors [47] and might control neuronal gene expression via calcineurin in distinct ways [48], conferring a new molecular phenotype with distinctive neuronal signalling.

Even though the structure of the P2X_4 _receptor in its closed state has been recently reported [49], the numerous serine residues in the N- and C-terminal domains of the P2X_3 _receptor make it difficult to predict those responsible for binding and catalytic activity of kinases and phosphatases. Future studies will be necessary to clarify this complex scenario in which multiple pathways control the phosphorylation state of P2X_3 _receptors with important and differential outcomes in terms of function and thus, presumably, in terms of pain sensing efficiency.

## Conclusions

Could P2X_3 _upregulation be an important component of migraine headache and to what extent do R192Q KI mice help our understanding of the disease process? The KI mouse model, carrying a human FHM-1 mutation, through abnormal activation of Ca_V_2.1 channels and a CaMKII-dependent pathway, exhibited potentiation of P2X_3 _receptor function in trigeminal neurons, thus suggesting the role of P2X_3 _receptors in trigeminal pain that requires future investigation especially because of the role of such receptors in sporadic migraine [13-16]. Because these purinergic nociceptors activate one major pain transducing mechanism of the trigeminovascular system [50], it is attractive to think that strong P2X_3 _receptor activation may facilitate ATP-dependent migraine headache [13,14]. Furthermore, the disabling headache attacks triggered by FK-506 treatment for immunosuppression in humans [51] might comprise a component due to intracellular Ca^2+ ^dysregulation and potential enhancement of P2X_3 _receptor function. Future studies are necessary to clarify the mechanisms responsible for transforming acute trigeminal pain into the long-lasting chronic headache typical of migraine attacks.

## Methods

### Genetic model

Ca_V_2.1 α1 R192Q mutant KI and wild type (WT) littermates were used for *in vitro *experiments. Genotyping was performed by PCR using primers 5'-TGTCGGGACGGAGTTTGAC-3' and 5'-AGACTCACGCACTTGGGATT-3' followed by enzyme digestion of PCR products with AlwNI (New England Biolabs, Ipswich, MA, USA) as previously described [5]. Animals were maintained in accordance with the Italian Animal Welfare Act and their use was approved by the Local Authority Veterinary Service.

### Culture of mouse trigeminal ganglia

**Figure 7:**
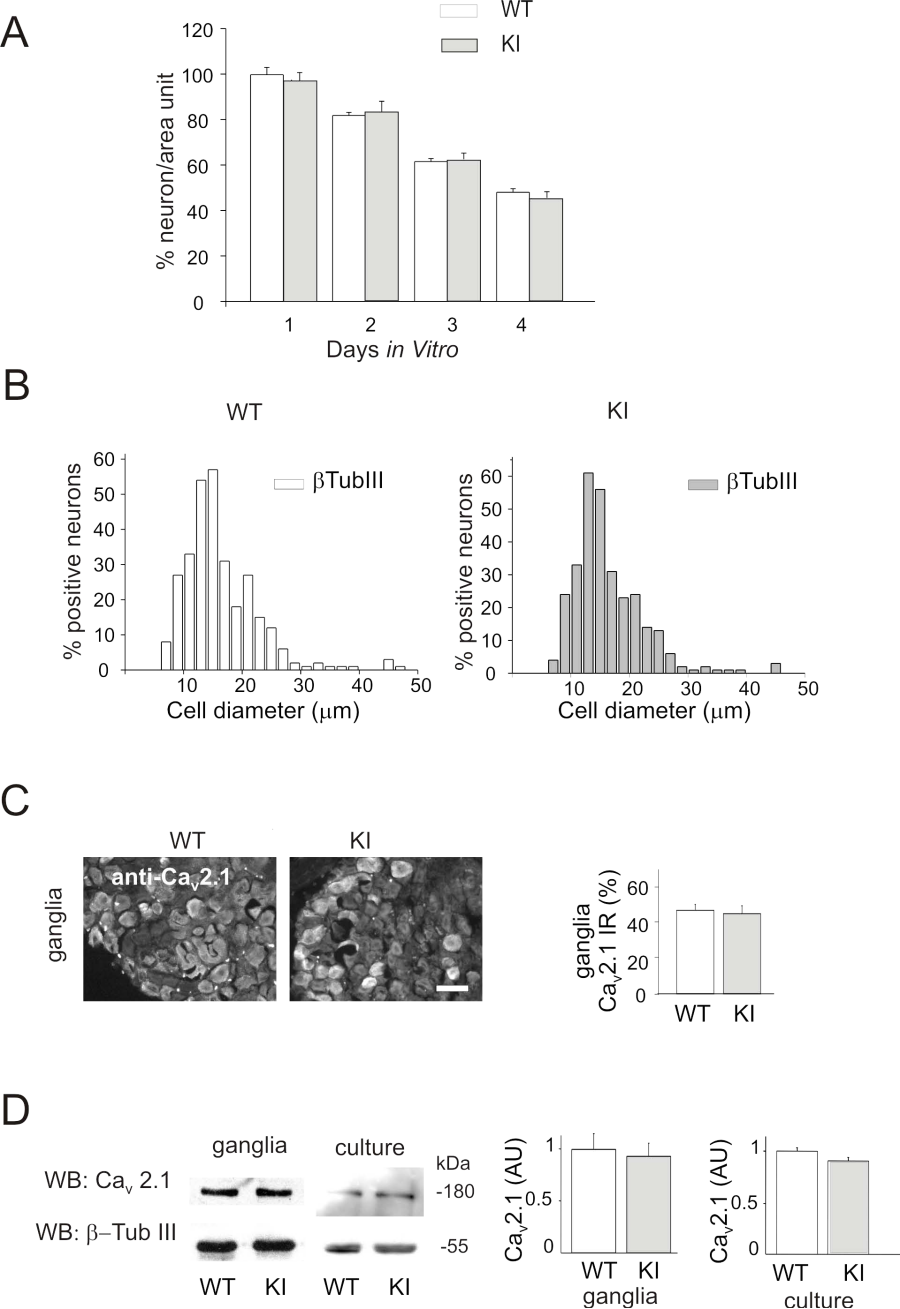
**Survival of WT and KI trigeminal neurons in culture and their expression of Ca**_**v**_**2.1 protein**. *A*, Survival is calculated as number of β-tubulin III positive cells per unit area after 1-4 days in culture. Data are normalized with respect to those at 1 day. *n *= 4, *p *> 0.05. *B*, Somatic size distribution of trigeminal neurons (β-tubulin III immunoreactive) in culture from WT and KI mice. *n *= 4. *C*, Immunocytochemical expression of Ca_V_2.1 channels in intact trigeminal ganglia of WT and KI mice. Histograms represent% of Ca_V_2.1 immunoreactive neurons over β-tubulinIII immunoreactive neurons in WT or KI ganglia. *n *= 3, *p *> 0.05. *D*, Example of western blot of protein extracts from WT and KI trigeminal ganglia or culture, probed with anti-Ca_V_2.1 antibody. Equal loading was ensured by membrane probing with β-tubulinIII antibodies. *n *= 3, *p *> 0.05. Histograms (*right*) show no significant difference between these conditions.

Trigeminal ganglion neurons were obtained from R192Q KI and WT mice (P12-P14) and cultured as described before [22]. Cells were used 24 h after plating. To support the comparison between KI and WT neurons, we ran initial feasibility studies to find out any obvious morphological or immunocytochemical differences between trigeminal neuronal cultures of R192Q KI and WT mice. Fig. 7A, B shows similar *in vitro *survival of neuronal subpopulations (*n *= 4, *p *> 0.05), whose largest fraction had somatic neuronal diameter of approximately 15 *μ*m with analogous distribution between genotypes for both ganglia and cultured ganglion neurons (Fig. 7B) in analogy with our previous observations [22]. We also confirmed that Ca_V_2.1 α1 expression as assessed by immunocytochemistry of intact trigeminal ganglia showed unchanged numbers of immunopositive cells in samples of both genotypes (Fig. 7C). Furthermore, Ca_V_2.1 α1 western blotting confirmed this finding and revealed similar expression levels in total membrane fractions from trigeminal neurons from intact ganglia or cultured ganglion neurons (Ca_V_2.1 α1 protein size: 180 kDa, *n *= 3, *p *> 0.05; Fig. 7D). The unchanged Ca_V_2.1 expression levels are in line with previous observations in the brains of R192Q KI mice [5].

The following compounds were added to the culture medium when appropriate: ω-agatoxin (200 nM), calcineurin inhibitor FK-506 (5 *μ*M), or the calcium calmodulin dependent kinase II (CaMKII) inhibitor KN-93 (5 *μ*M). These compounds were pre-applied for 60-90 min or overnight as indicated. All compounds were obtained from Sigma (Milan, Italy).

### Immunofluorescence microscopy

For immunofluorescence microscopy, the following antibodies were used: anti-P2X_3_, anti-P2X_2_, anti-TRPV1 and anti-Ca_V_2.1 α1 (1:200; all from Alomone Labs, Jerusalem, Israel); anti-P2X_3 _(1:300; Neuromics, Edina, MN, USA), anti-β-tubulin III (1:1000; Sigma), anti-phosphorylated Thr286 CaMKII (1:500; Promega, Madison, WI, USA), anti-phosphorylated Ser133 CREB (1:300; Upstate Millipore, NY, USA). Antibodies were incubated for 2 h in phosphate saline buffer plus 5% bovine serum albumin and 0.1% Tween20. Secondary antibodies anti-rabbit, anti-mouse or anti-guinea pig conjugated with AlexaFluor488 or AlexaFluor594 were from Invitrogen (1:500; S. Giuliano Milanese, Italy). Staining with secondary antibodies only were performed as control experiments and did not give a signal. Stained sections were analysed with a Zeiss microscope and MetaMorph software (Molecular Devices, Downingtown, PA, USA). On average, 200 cells were analyzed in each test; data are the mean of at least 3 independent experiments.

### Protein lysates, immunoprecipitation and immunoblotting

For western blotting and immunoprecipitation experiments, proteins from ganglia or cultures were extracted in TNE buffer (10 mM Tris-HCl at pH 7.5, 150 mM NaCl, 2 mM EDTA, 100 mM NaF, 2% n-octyl β-D-glucopyranoside and 1% NP40) plus protease inhibitors (Sigma). Membrane extracts were performed as described [23]. The following antibodies were used: anti-P2X_3 _(1:300; Alomone Labs), anti-P2X_3 _(H-60, 1:500; Santa Cruz Biotechnology, Inc., Santa Cruz, CA, USA); anti-cyclin dependent kinase 5 (Cdk5; 1: 400; Santa Cruz), anti-p35 (1:200, Santa Cruz), anti-Ca_V_2.1 α1 (1:200; Alomone Labs), anti-phosphorylated tyrosine HRP-conjugated clone Y20 (1:3,000; Invitrogen), anti-phospho-serine (1:600; Millipore), anti-phosphorylated threonine (1:600; Cell Signaling Danvers, MA, USA), anti-β-tubulin III (1:2,000; Sigma), anti-actin (1:3,000: Sigma). To avoid complications in the analysis because of the presence of immunoglobulin heavy chains, a mouse anti-rabbit IgG-HRP conjugated (Jackson ImmunoResearch, Suffolk, UK) was used as secondary antibody. Western blot signals were detected with the enhanced chemiluminescence light system (GE Healthcare, Milano, Italy). For quantification, band density was measured using CorelDraw Photopaint software and ImageJ software. Membrane protein biotinylation and streptavidin pulldown experiments were performed, as described previously [9].

### Real time RT-PCR

Total mRNA extraction from trigeminal cultures or ganglia and reverse transcription reactions was performed as described previously [9]. Data were normalized with respect to β-tubulin III housekeeping mRNA contents using primers previously reported [9].

### Patch-clamp recording

After 1 day in culture, trigeminal neurons were superfused continuously (2 mL/min) with physiological solution containing (in mM): 152 NaCl, 5 KCl, 1 MgCl_2_, 2 CaCl_2_, 10 glucose, and 10 HEPES (pH adjusted to 7.4 with NaOH). Cells were patch-clamped in the whole-cell configuration using pipettes with a resistance of 3-4 MΩ when filled with the following solution (in mM): 140 KCl, 0.5 CaCl_2_, 2 MgCl_2_, 2 Mg_2_ATP_3_, 2 GTP, 10 HEPES, and 10 EGTA (pH adjusted to 7.2 with KOH). Cells were held at -60 mV. Currents were filtered at 1 kHz and acquired by means of a DigiData 1200 Interface and pClamp 8.2 software (Molecular Devices, Sunnyvale, CA, USA). To obtain stable and reproducible P2X_3 _receptor currents, its synthetic agonist α,β-methylene-adenosine-5'-triphosphate (α,β-meATP) was applied using a fast superfusion system (Rapid Solution Changer RSC-200; BioLogic Science Instruments, Claix, France). The time for solution exchange was approximately 30 ms. Responses were measured as peak amplitude, while the response rise time was expressed as its time constant (τ_on_). Whenever the current response did not decay to baseline prior the end of agonist application, we considered the residual current as indicative of heteromeric P2X_2/3 _receptors [21] and measured it to quantify its size and occurrence. To express agonist potency as EC_50 _values (concentration producing 50% of the maximum response), dose-response curves were constructed by applying different agonist doses to the same cells, and fitting them with a logistic equation (Origin 6.0; Microcal, Northampton, MA, USA). The onset of desensitization was estimated by calculating the first-time constant of current decay (τ_fast_) in accordance with our previous reports [52]. Previous studies have indicated that recovery from desensitization of P2X_3 _receptors is a relatively slow process which depends on the agonist used for the test [52,53]. In the case of α,β-meATP, the half-time recovery is typically under 1 min and thus, we use the amplitude of a second response evoked by α,β-meATP 30 s after the first one as a simple index to assess the extent of recovery from desensitization in accordance with former reports [9,22]. Heteromeric responses of native P2X_2/3 _receptors were identified on the basis of their residual current (≥5% of peak current) at the end of a 2-s-long agonist application as previously reported [9]. Capsaicin was applied at the standard test dose of 1 *μ*M (for 2 s) to evoke reproducible inward currents. Functional current responses were recorded approximately 10 min after washout of the culture medium. To quantify the effect of a drug on the α,β-meATP-induced current amplitude for a drug-treated neuron, the peak response was expressed as a percentage of the mean peak current amplitude obtained from control neurons from cultures prepared in parallel.

### Ca^2+ ^imaging

Cultured trigeminal neurons were incubated for 40 min at 20-22°C in physiological solution containing Fluo3-AM (5 *μ*M; Invitrogen) followed by a wash for 30 min. Fluorescence emission signals were acquired with a CCD camera (Coolsnap HQ; Roper Scientific, Duluth, GA, USA). We did not observe any difference in dye loading between WT and KI neurons. Data were collected from cells that produced a rapid response to a pulse of high concentration of KCl (i.e., 50 mM for 1 s) to maximize the activation of voltage-dependent Ca^2+ ^channels: their responsiveness indicated that they were neurons [10]. We could not detect any difference in the amplitude of such responses between WT and KI neurons tested in parallel with sister cultures. For routine testing of the neuronal excitability we used 20 mM K^+ ^to stimulate neurons without excessive depolarization and 10 *μ*M α,β-meATP in analogy to patch clamp protocols. The analysis was performed using Metafluor software (Metafluor Imaging Series 6.0; Molecular Devices). Intracellular Ca^2+ ^transients are expressed as fractional amplitude increase (Δ*F*/*F*_0_, where *F*_0 _is the baseline fluorescence level and Δ*F *is the increment over baseline). To test the effect of ω-agatoxin (200 nM) on Ca^2+ ^transients, neurons were challenged with a 2-s pulse of α,β-meATP, capsaicin, 20 mM K^+^, 50 mM K^+ ^before and after 30 min application of ω-agatoxin.

### Pharmacological inhibitors

Pharmacological blockers were used to support our biochemical and electrophysiological data suggesting a role for certain enzymes and channels in the observed mouse neuron phenotype. In particular, we used ω-agatoxin (200 nM) as a selective inhibitor of P/Q-type Ca^2+ ^channels [54]: this toxin has been extensively employed to probe the influence of such channels on a variety of in vivo pain models [55,56] including trigeminal neurons [57]. To block activated CaMKII, we used KN-93 (5 μM; see also [11]), an agent widely employed to depress experimental pain [58-60]. Conversely, we also applied FK-506 (5 μM), a well known immunosuppressant drug that acts by binding to proteins regulating the Ca^2+^-dependent phosphatase calcineurin [39]. This drug can induce strong, persistent pain in humans [61], including severe headache [51]. We also tested the calcineurin autoinhibitory peptide F2/47 (100 μM; Tocris, Bristol, UK) [40] applied via the intracellular solution in patch clamp studies.

### Statistics

Data are expressed as mean ± standard error of the mean (SEM), where *n *indicates the number of experiments in molecular biology/immunocytochemistry or the number of investigated cells in electrophysiology (unless stated otherwise). Statistical analysis was performed using the Student's *t*-test, the Mann-Whitney rank sum test or the ANOVA test, when appropriate for parametric or non-parametric data, respectively. A *p *value of ≤ 0.05 was accepted as indicative of a statistically significant difference.

## Abbreviations

α,β-meATP: α,β-methyleneadenosine 5'-triphosphate; Ca_v_2.1: voltage activated calcium channel 2.1; CaMKII: calcium/calmodulin dependent kinase II; Cdk5: cyclin dependent kinase 5; CSD: cortical spreading depression; EC_50_: effective concentration 50; FHM-1: familial hemiplegic migraine type 1; KI: knock-in; P2X_2_: purinergic ionotropic receptor 2; P2X_3_: purinergic ionotropic receptor 3; P2X_2/3_: heteromeric P2X_2/3 _receptor; PP1: protein phosphatase 1; PP2A: protein phosphatase 2A; PP2B: protein phosphatase 2B/calcineurin; PP2C: protein phosphatase 2C; RT-PCR: real time polymerase chain reaction; TG: trigeminal ganglia; TRPV1: transient potential receptor vanilloid 1; WT: wild type.

## Competing interests

The authors declare that they have no competing interests.

## Authors' contributions

All authors read and approved the final manuscript. ANair and MS provided equal contribution to this study.  ANair and RG, design and collection of functional data; MS and NB, collection of molecular data; MDF and AvdM, design and supply of genetic model; ANistri, supervision and design of functional studies; EF, supervision and design of molecular studies; ANistri, AvdM and EF, joint contribution to manuscript writing.
